# Heme Dissociation from Myoglobin in the Presence of the Zwitterionic Detergent *N*,*N*-Dimethyl-*N*-Dodecylglycine Betaine: Effects of Ionic Liquids

**DOI:** 10.3390/biom8040126

**Published:** 2018-10-29

**Authors:** Eric M. Kohn, Joshua Y. Lee, Anthony Calabro, Timothy D. Vaden, Gregory A. Caputo

**Affiliations:** 1 Department of Chemistry and Biochemistry, Rowan University, Glassboro, NJ 08028, USA; kohne2@students.rowan.edu (E.M.K.); leej8@students.rowan.edu (J.Y.L.); calabroa6@students.rowan.edu (A.C.); vadent@rowan.edu (T.D.V.); 2 Bantivoglio Honors College, Rowan University, Glassboro, NJ 08028, USA; 3 Department of Molecular & Cellular Biosciences, Rowan University, Glassboro, NJ 08028, USA

**Keywords:** ionic liquids, myoglobin, detergents, protein folding

## Abstract

We have investigated myoglobin protein denaturation using the zwitterionic detergent Empigen BB (EBB, *N*,*N*-Dimethyl-*N*-dodecylglycine betaine). A combination of absorbance, fluorescence, and circular dichroism spectroscopic measurements elucidated the protein denaturation and heme dissociation from myoglobin. The results indicated that Empigen BB was not able to fully denature the myoglobin structure, but apparently can induce the dissociation of the heme group from the protein. This provides a way to estimate the heme binding free energy, ΔG_dissociation_. As ionic liquids (ILs) have been shown to perturb the myoglobin protein, we have investigated the effects of the ILs 1-butyl-3-methylimidazolium chloride (BMICl), 1-ethyl-3-methylimidazolium acetate (EMIAc), and 1-butyl-3-methylimidazolium tetrafluoroborate (BMIBF_4_) in aqueous solution on the ΔG_dissociation_ values. Absorbance experiments show the ILs had minimal effect on ΔG_dissociation_ values when compared to controls. Fluorescence and circular dichroism data confirm the ILs have no effect on heme dissociation, demonstrating that low concentrations ILs do not impact the heme dissociation from the protein and do not significantly denature myoglobin on their own or in combination with EBB. These results provide important data for future studies of the mechanism of IL-mediated protein stabilization/destabilization and biocompatibility studies.

## 1. Introduction

It has proven very difficult to directly investigate the thermodynamics and kinetics of native protein folding due to the fact that proteins fold in the cell, often co-translationally. Many natural proteins also require the action of chaperones to properly fold into the functional three-dimensional (3D) conformation. The folding process in cells also is directly impacted by the incorporation of non-protein cofactors and prosthetic groups such as heme. However, only very elegant experimental designs using whole cell extracts and non-native amino acids can begin to address folding in a cellular context [1]. As such, the primary approach to studying protein structural stability has been to use unfolding or denaturation studies using chemical denaturants or thermal energy to unfold the protein. Numerous chemical denaturing agents such as guanidinium chloride, urea, and detergents have been commonly employed to unfold proteins. Denaturants often cause protein destabilization through different molecular mechanisms such as H-bonding to backbone amides and carbonyls [2,3], disruption of ionic interactions [4], or through hydrophobic intercalation [5,6,7], thereby disrupting secondary and/or tertiary structure. However, the specific mechanism of protein denaturation depends on the denaturing agents [8,9].

The use of surfactants or detergents to denature proteins is widely applied in life sciences research, most ubiquitously in the use of SDS-PAGE (Sodium Dodecyl Sulfate—PolyAcrylamide Gel Electrophoresis) [10,11]. Detergents denature proteins primarily by interdigitating the hydrophobic acyl chains in the interior of folded proteins, disrupting hydrophobic contacts, which leads to a destabilization of the folded structure. However, there is significant variation in the fine-grained mechanism of denaturation which has been shown to be dependent on detergent charge, micelle size, sample pH, critical micelle concentration (CMC), protein structure, and protein composition [7,12,13,14]. Nonetheless, the majority of reports in the literature have focused on the use of either cationic or anionic detergents such as tetradecyltrimethylammonium bromide (TTAB) and sodium dodecyl sulfate (SDS), with very few examples of protein denaturation carried out using zwitterionic or nonionic detergents.

Protein unfolding studies in the presence of ionic liquids (ILs) have recently gained attention in order to understand the effects of ILs on protein structures and stabilities for their potential biomedical applications. The tunable properties of the cation-anion pairs in ILs allow for selection of compatibility and optimal functionality for a variety of desired applications. There have been a variety of reports describing the application of ILs for enzyme inhibition, drug delivery, and biomedical devices [15,16,17,18,19,20,21,22,23], and their cellular compatibility. In addition to the biomedical and life science application studies, the fundamental properties of ILs, as well as their properties in the presence of biomolecules, have been an area of extensive research. Ionic liquids have been demonstrated to exhibit properties similar to typical electrolytic salts following the Hofmeister series when combined in aqueous solution with proteins [24,25,26,27,28]. Depending on the structure of the cation-anion molecules, ILs have also exhibited the ability to phase-partition between organic and aqueous phases, or to aggregate and micellize in a manner similar to detergents [19,21,29,30,31,32,33,34,35,36]. The micellization activity is primarily driven through the alkyl chains attached to the cation component of the IL [35].

Numerous recent studies, both experimental and computational, have focused on the interactions of ILs with proteins [11,26,30,33]. Many groups have investigated the interactions of different ILs with industrially relevant proteins and enzymes such as laccase [34], thermolysin [29], lipase [37], and as additives to promote protein crystallization [38,39]. In addition, many ILs have been investigated with respect to their ability to stabilize or destabilize protein structures [40]. Recently, 1-ethyl-3-methylimidazolium ethylsulfate (EmimESO_4_) was shown to enhance the thermal stability of bovine serum albumin (BSA) [33], while a study focused on the role of the anionic IL component demonstrated 1-Butyl-3-methylimidazolium Nitrate (BMINO_3_)was more destabilizing than the analogous 1-butyl-3-methylimidazolium chloride (BMICl) [41].

**Figure 1:**
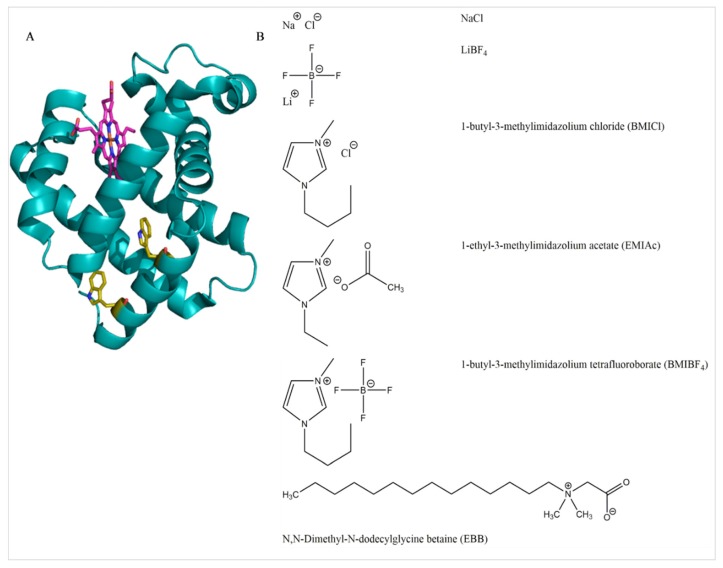
Molecular structures (**A**) 3D structure of myoglobin from PDBID: 1WLA. The heme group is colored magenta while the two native Trp residues are yellow. (**B**) Chemical structures of salts, ionic liquids (ILs), and the Empigen BB (EBB) detergent.

In this work, the combination of several ILs (BMIBF_4_, BMICl, EMIAc) and control salts (LiBF_4_, NaCl) with the zwitterionic detergent Empigen BB (EBB) and the effects of these combinations on the oxygen storage protein myoglobin (Figure 1) was investigated. Myoglobin has proven to be a good model system for studying protein denaturation due to high solubility, uniformly α-helical structure, and visible absorbance signature arising from the heme cofactor [5,42,43]. Additionally, the conversion of myoglobin between apo- to holo- forms can be monitored by the same spectroscopic approaches. The choice of detergent was driven by the fact that EBB is used in the purification/stabilization of some proteins [44,45] and is zwitterionic, thus decreasing possible effects due to ionic interactions with the ILs. We utilize conventional spectroscopic approaches including ultraviolet (UV)-vis absorbance, fluorescence, and circular dichroism (CD) to monitor the unfolding of the myoglobin by EBB in the presence of differing concentrations of IL. We also performed the control experiments to ensure that the presence of ILs was not significantly impacting the CMC of EBB which would potentially influence the unfolding behavior. The EBB is shown to induce minimal structural denaturation but does induce the loss of the heme group (holo- to apo-conversion). This resulted in a loss of absorptivity in the Soret band of the heme, loss of a CD signature around the same band, and a relief of iron/heme-based heavy-atom quenching of native Trp residues in the protein [42,43]. The results presented show that the EBB does not completely unfold the protein but does likely remove the heme group, and the effect of ILs on this process is negligible. 

## 2. Materials and Methods

### 2.1. Materials

Horse skeletal myoglobin (95–100% lyophilized powder) and Empigen BB (Empigen BB ~30% pure liquid) were purchased from Sigma Life Sciences (St. Louis, MO, USA); myoglobin was stored at −20 °C in the dark. BMIBF_4_, BMICl, EMIAc, LiBF_4_ and 1,6-Diphenyl-1,3,5-hexatriene (DPH) were purchased from Sigma Chemical Company (St. Louis, MO, USA). Ionic liquids were >97% purity. NaCl was purchased from Fisher Scientific (Fair Lawn, NJ, USA).

### 2.2. Sample Preparation

All experiments were carried out in 2 mM sodium phosphate buffer (pH 6.9–7.2) using aqueous solutions of myoglobin, ILs, and Empigen BB in deionized water. Final myoglobin concentrations were kept at 0.15 mg/mL for all experiments excluding those examined by CD spectroscopy in the far UV region; for these experiments, myoglobin concentrations were decreased to 0.0375 mg/mL (by a factor of four) as the original 0.15 mg/mL solutions produced a signal greater than the detection range of the instrument used. Each sample contained one species of ionic liquid at a concentration of 0, 9.4, 28.1 or 56.3 mM. For absorbance and fluorescence experiments, unfolding was monitored by performing experiments in triplicate for all species and concentrations of ionic liquid 0.00, 0.10, 0.20, 0.30, 0.40, 0.50, 0.75, 1.00, 1.25, 1.50, or 1.75 mM EBB. This was achieved by adding 25 mM EBB in increasing volumes across samples. The appropriate volume of phosphate buffer was added to bring the volume of each sample to 1000 μL.

### 2.3. Critical Micelle Concentration Determination

Micelle formation assays were conducted using stock solutions of 1.5 M NaCl, LiBF_4_, BMIBF_4_, and EMIAc in water as well as 0.4 mM DPH solution, a 16 mM solution of EBB and a 2 mM sodium phosphate buffer solution, with a pH 6.9–7.2. The samples contained 0 mM, 50 mM, 100 mM, 150 mM or 300 mM of salt or ionic liquid, 2.5 µm DPH, and a serially diluted concentration of detergent ranging from 0.125–8 mM. Assays were performed in 384-well plates and DPH fluorescence was measured using a Molecular Devices SpectraMax i3x plate reader (Sunnyvale, CA, USA) with an excitation of 358 nm and an emission of 430 nm. Each datum is the average and standard deviation from 12 replicates. The data was plotted in excel and then analyzed for the CMC using linear fits to determine the onset of micelle formation and the plateau after micelle formation using an *R*^2^ cutoff of 0.9. The midpoint of the fluorescence intensity transition was determined by fitting the data to cubic polynomial and subsequently taking the derivative to determine the inflection point.

The fluorescence emission spectra of DPH in the presence of EBB and without EBB as well as with and without the controls and ionic liquids were also measured. Samples contained 300 mM salt or ionic liquid, 8 mM detergent, 1.25 µM DPH in phosphate buffer. Fluorescence emission spectra were measured using a Horiba Scientific FluoroMax-4 Spectrofluorometer (Edison, NJ, USA) λ_ex_ = 358 nm, λ_em_ = 380–680 nm.

### 2.4. Absorbance Measurement

Myoglobin, ILs, and EBB were combined in buffer and allowed incubate for 30 min at room temperature. Samples were prepared in polystyrene cuvettes (optically clear to 340 nm) to measure absorbance spectra from 375–575 nm or in quartz cuvettes to measure absorbance spectra from 200–700 nm. Absorbance spectra were recorded using a Thermo Scientific Genesys 10S UV-Vis Spectrophotometer (Waltham, MA, USA). Dissociation of heme (*H*) was calculated at 409 nm by

(1) $$H=\frac{A-A_{i}}{A_{f}-A_{i}},$$

where *A* is the absorbance of a sample at a given concentration of EBB, *A_i_* represents the maximum heme absorbance, i.e., 100% bound (0 mM EBB), and *A_f_* represents the minimum amount of bound heme (1.75 mM EBB).

### 2.5. Fluorescence Spectroscopy

Fluorescence quenching was measured using a SpectraMax M5 or i3x plate reader (Molecular Devices) in clear, untreated UV-Star 96-well plates purchased from Greiner Bio One (655801, Kremsmünster, Upper Austria) with an optical window down to 230 nm. The excitation and emission were at 280 nm and 350 nm, respectively. The dissociation of heme (*H*) was similarly calculated at 350 nm by

(2) $$H=\frac{I-I_{i}}{I_{f}-I_{i}}$$

for each sample, where *I* is the fluorescence emission intensity at a given concentration of EBB, *I_i_* represents the maximum heme emission intensity, i.e., 100% bound (0 mM EBB), and *I_f_* represents the minimum amount of bound heme (1.75 mM EBB).

Fluorescence emission spectra were collected in triplicate for samples containing 0 or 30 mM NaCl, BMICl, or BMIBF_4_; 0 or 1.75 mM EBB; and 0 or 0.15 mg/mL myoglobin in quartz semi-micro cuvettes with a Fluoromax-4 Spectrophotometer (Horiba Scientific).

### 2.6. Circular Dichroism Spectroscopy

Circular dichroism was performed using a CD spectropolarimeter model J-810 by Jasco (Easton, MD, USA) for each concentration and species of ionic liquid, at all concentrations of EBB. Spectra were recorded between 190–260 nm to assess secondary structure and 375–500 nm to monitor dissociation of heme; from this, the signals produced under 408–410 nm light were averaged. Background correction was performed by subtracting the signal produced by each species and concentration of ionic liquid in buffer from those of the samples containing myoglobin and EBB.

### 2.7. Calculation of Gibbs Free Energy of Dissociation

The equilibrium constant of dissociation [*K_eq_*] in each ionic liquid environment was calculated by

(3) $$K_{eq}=-RTln\left(\frac{OD_{409}}{1-OD_{409}}\right),$$

where *R* is the ideal gas constant (8.314 J·mol^−1^), *T* is the temperature in kelvin (295 K was estimated for all experiments), and *OD*_409_ is the peak absorbance of myoglobin solution at 409 nm. Data were analyzed in Microsoft Excel (Redmond, WA, USA) and fit to a linear trendline with an *R*^2^ cutoff of 0.95. The y-intercept of each trendline was the estimated Gibbs free energy of dissociation (ΔG_dissociation_). This value reflects the energy of dissociation of the heme group from the myoglobin protein. The ΔG_dissociation_ values were tested for statistical significance using an analysis of variance (ANOVA) and post-hoc testing for each variable using MS Excel with the XL-toolbox add-in [46].

Interventionary studies involving animals or humans, and other studies require ethical approval must list the authority that provided approval and the corresponding ethical approval code.

## 3. Results

### 3.1. Protein Denaturation and Heme Dissociation by Empigen BB

**Figure 2:**
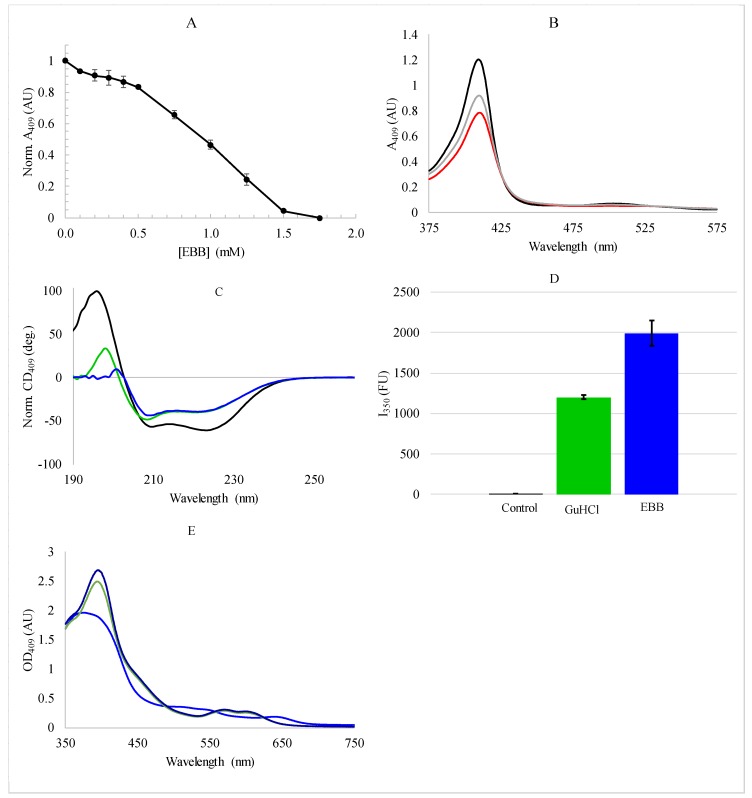
Spectroscopic analysis of myoglobin unfolding. (**A**) Heme absorbance at 409 nm (*A*_409_) as a function of EBB concentration; (**B**) Absorbance spectra of myoglobin with 0 mM EBB (black), 1 mM EBB (gray), or 1.75 mM EBB (red); (**C**) Representative circular dichroism (CD) spectra for myoglobin with: 0 mM EBB (black), 1.75 mM EBB (green), 8 mM EBB (blue); (**D**) Fluorescence intensity (*I*) of Trp emission from myoglobin in buffer (black), denatured with 1.75 mM EBB (blue), or denatured with 3 M GuHCl (green). Fluorescence data are the averages and standard deviations of five samples; (**E**) Absorbance spectra of 50 µM heme B in phosphate buffer (black) or buffer supplemented with 1.75 mM EBB (green) or 8 mM EBB (blue). All samples were in 2 mM sodium phosphate, pH 7.

Empigen BB is not a typical denaturant for proteins, and therefore it is important to characterize the interaction of this zwitterionic detergent with myoglobin and heme. As absorbance spectroscopy in the heme region is typically used to quantify myoglobin unfolding [13,42], the absorbance at 409 nm was monitored as a function of detergent concentration (Figure 2A,B). While there is a significant reduction in the heme Soret band at 409 nm (Figure 2A), this band does not disappear. However, the absorbance profile is reminiscent of a standard protein unfolding curve (Figure 2B). To ascertain whether or not Figure 2A,B indicate protein unfolding, the myoglobin secondary structure was directly characterized in the presence of EBB using CD spectroscopy in the mid-UV region (Figure 2C). Notably, there is only a minor loss of helical content upon addition of 1.75 mM EBB (~15%) which is only slightly enhanced by increasing detergent concentration up to 8 mM (Figure 2C, and see below—discussion). The CD results indicate that EBB does not fully denature the protein and therefore the loss of absorbance at 409 nm is likely due more to the dissociation of the heme group from the myoglobin than protein denaturation.

In light of these findings, we compared heme fluorescence dequenching when myoglobin was denatured by EBB with denaturation by the chaotrope guanidinium chloride (GuHCl). It has been well-demonstrated in the literature that as the distance between the native Trp residues and the heme increases, Trp fluorescence emission intensity increases. Our fluorescence results (Figure 2D) show that the Trp fluorescence significantly increases both when the protein is completely denatured by GuHCl and when it is partially denatured by EBB, indicating that the Trp-heme distance is large in both cases. These results indicate that while the protein is not completely denatured by EBB (based on the CD results), the heme is clearly removed from the protein binding pocket under these conditions. For control, we measured the absorbance of the free heme molecule in solutions with and without EBB (Figure 2E). These spectra show a similar bathochromic shift in peak position as the heme traditionally exhibits in the denaturation of myoglobin [13,42], but with different magnitude shifts relative to literature values. The heme absorbance decreases slightly with 1.75 mM EBB (and more so with 8 mM EBB).

### 3.2. Spectroscopic Analysis of Heme Dissociation from Myoglobin with Ionic Liquids

Considering EBB does not cause full denaturation of the protein but is sufficient to remove the heme, the effect of low concentrations of ILs on this process was monitored using absorbance spectroscopy, specifically using the heme absorption, or Soret band, at 409 nm. As the heme is lost from the binding pocket there is a concomitant loss of absorptivity and a slight red shift in the Soret band [42,43,47]. Control experiments indicated that the effects were complete in under 10 min of incubation with the detergent, so all samples were allowed to equilibrate with the Empigen BB for 30 min before measurement to ensure equilibrium was achieved. The absorbance of 0 mM detergent sample was normalized to 100% bound and the fraction of bound/unbound heme was calculated from the absorbance measurements similar to previously reported [42,43].

**Figure 3:**
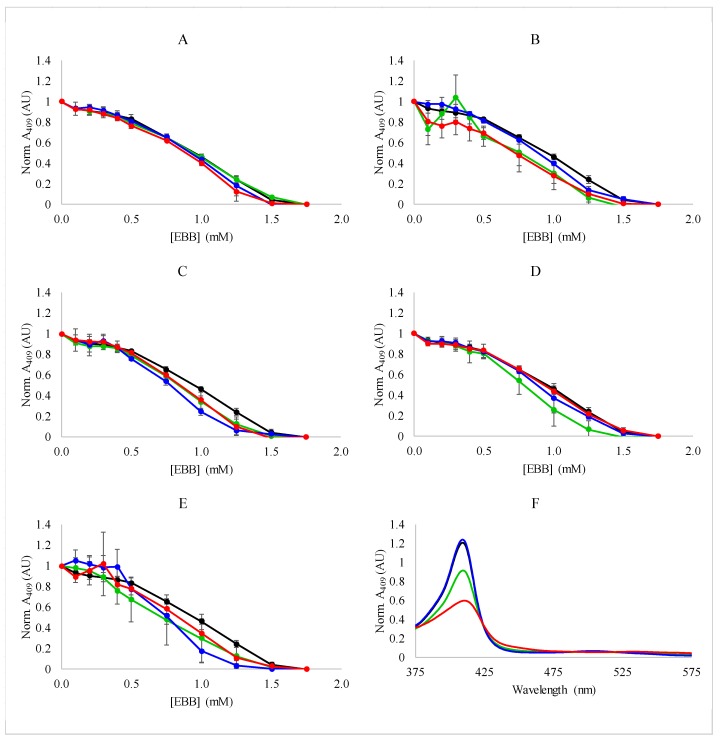
Dissociation of heme from myoglobin monitored by absorbance spectroscopy—Denaturation of myoglobin (0.15 mg/mL) by EBB was monitored by the loss of heme absorbance at 409 nm. Experiments were performed in the presence of 0 (black), 9.4 mM (green), 28.1 mM (blue), 56.3 mM (red) ionic liquids or salts. (**A**) NaCl; (**B**) LiBF_4_; (**C**) BMICl; (**D**) EMIAc; (**E**) BMIBF_4_. Normalization was performed by setting the absorbance at 409 nm to 1 at 0 detergent concentration. Panel (**F**) shows representative absorbance spectra for myoglobin with: 0 mM BMIBF4 0 mM EBB (black), 0 mM BMIBF_4_ 1.75 mM EBB (green), 56.3 mM BMIBF_4_ 0 mM EBB (blue), 56.3 mM BMIBF_4_ 1.75 mM EBB (red). All data in panels (**A**–**E**) are averages of three independent samples and error bars represent the standard deviation of the replicates.

Myoglobin was exposed to increasing concentrations of Empigen BB in the presence of a fixed concentration of IL or salt. The results of these experiments are shown in Figure 3. The results demonstrate several important points. First, the EBB removes the heme with a typical sigmoidal, two state transition that is reminiscent of myoglobin denaturation by SDS and other denaturants [48]. It is important to note that the results from experiments shown in Figure 2 indicate this transition is more likely the result of heme dissociation, not denaturation. With respect to IL/salt concentration, increasing the concentration of the ionic additive did not cause significant shifts in the dissociation curves, indicating the process is generally insensitive to the ionic strength of the solution. Most interestingly, the IL BMIBF_4_ did not significantly affect the dissociation profile with EBB. This is an important finding as it was previously shown that high concentrations of BMIBF_4_ can denature myoglobin in the absence of other chaotropes [42,43]. However, the absorbance spectrum of the samples containing detergent and BMIBF_4_ exhibited a lower absorbance than the samples treated with detergent alone (Figure 3F). This supports the idea that the destabilizing effect of BMIBF_4_ is concentration dependent, and that myoglobin folding is insensitive to low concentrations of BMIBF_4_, however, there may be additional BMIBF_4_-heme interactions that affect absorptivity but not the folding stability of the protein. These absorbance spectroscopy results were also utilized to determine the ΔG_dissociation_ associated with the EBB induced dissociation of heme (see below).

The EBB-induced heme dissociation was next investigated by changes in emission intensity of the intrinsic tryptophan. In the folded state, the iron at the center of the heme group efficiently quenches Trp fluorescence emission through a heavy atom quenching mechanism [49,50]. As the protein unfolds, the Trp-heme distance increases as the heme is lost, relieving the quenching effect, resulting in an increase in Trp emission intensity. Based on the crystal structure of myoglobin (PDBID 1WLA), in the folded state the two native Trp residues are ~23 Å and ~16 Å from the heme (measured from heme iron to Trp indole nitrogen). This dequenching assay can be more sensitive to subtle changes in folding or to heme dissociation as even small changes in distance between quencher and fluorophore can have significant effects on quenching efficiency. The results in Figure 2 show that when myoglobin is exposed to EBB, the dissociation of heme is sufficient to relieve the heme-Trp quenching.

**Figure 4:**
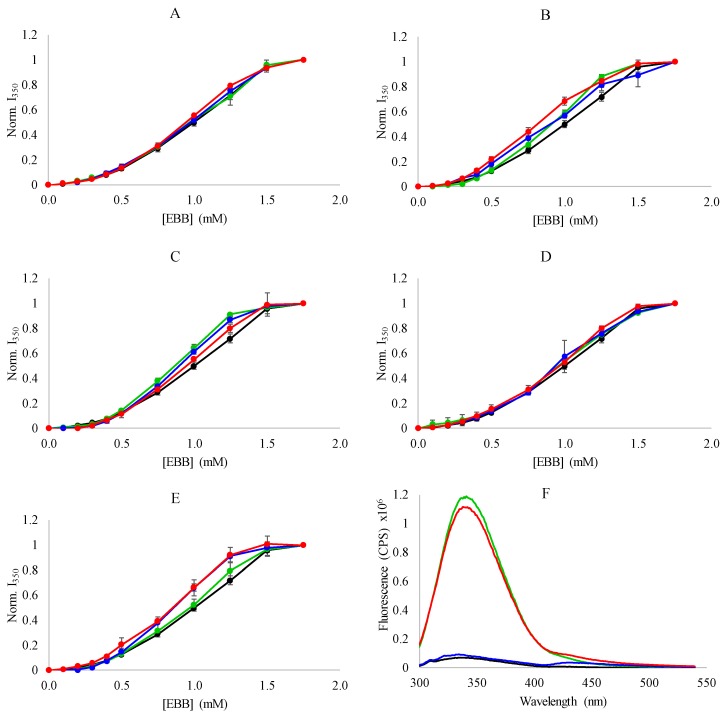
Dissociation of heme monitored by fluorescence spectroscopy—Denaturation of myoglobin (0.15 mg/mL) by EBB was monitored by the relief of heme quenching of native Trp fluorescence at 350 nm by when excited by 280 nm light. Experiments were performed in the presence of 0 (black), 9.4 mM (green), 28.1 mM (blue), 56.3 mM (red) ionic liquids or salts. (**A**) NaCl; (**B**) LiBF_4_; (**C**) BMICl; (**D**) EMIAc; or (**E**) BMIBF_4_. Normalization was performed by setting the fluorescence at 350 nm to 1 at 0 detergent concentration. Panel (**F**) shows representative fluorescence spectra for myoglobin with: 0 mM BMIBF_4_ 0 mM EBB (black), 0 mM BMIBF_4_ 1.75 mM EBB (green), 56.3 mM BMIBF_4_ 0 mM EBB (blue), and 56.3 mM BMIBF_4_ 1.75 mM EBB (red). All data in panels (**A**–**E**) are averages of three independent samples and error bars represent the standard deviations of the replicates.

The results of the dissociation experiments monitored by Trp fluorescence are shown in Figure 4. The Trp intensity increases upon addition of EBB indicating that the distance between the heme and the Trp residues is increasing. Again, as was observed for the absorbance measurements, the Trp intensity increases are similar in profile between samples with no salt/IL and those with 9.4 mM, 28.1 mM, and 56.3 mM IL/salt included in the sample. These results indicate that treatment with Empigen BB results in a final conformation in which the heme is significantly far from the native Trp residues.

Circular dichroism spectroscopy is a widely utilized method for evaluating protein folding because of the distinctive spectral shapes associated with protein secondary structures in the far UV spectral region. However, due to the strong absorption of the imidazolium-based ILs in the far UV region, traditional protein structural analysis by CD is unreliable. For this reason, the signal produced by heme was used, as the restricted chiral environment provided by myoglobin generates a positive CD band to protein-associated heme [42]. This approach can also be used to investigate the removal or dissociation of the heme group from the myoglobin protein, as the removal of heme from the binding pocket decreases this signal regardless of protein secondary structure or loss thereof.

**Figure 5:**
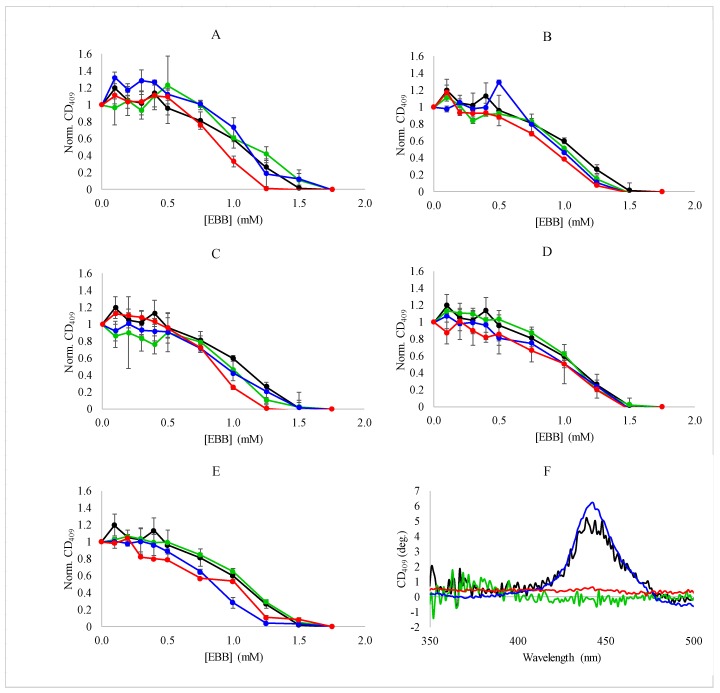
Denaturation of myoglobin monitored by CD spectroscopy—Denaturation of myoglobin (0.15 mg/mL) by EBB was monitored by change in CD signal around the heme transition. Experiments were performed in the presence of 0 (black), 9.4 mM (green), 28.1 mM (blue), or 56.3 mM (red). (**A**) NaCl; (**B**) LiBF_4_; (**C**) BMICl; (**D**) EMIAc; or (**E**) BMIBF_4_. Normalization was performed by setting the CD signal at 409 nm to 1 at 0 detergent concentration. Panel (**F**) shows representative CD spectra for myoglobin with (green) and without EBB (black) in buffer or with (red) or without (blue) EBB in the presence of 56.3 mM BMIBF_4_.

Heme dissociation was also monitored by the loss of the CD signal at 409 nm as a function of EBB in the presence of varying concentrations of ILs or salts. Similar to the results from the absorbance measurements, the CD results showed the sigmoidal dissociation profiles (Figure 5). Consistent with the absorbance and fluorescence measurements, the ILs and salts did not have a dramatic impact on the dissociation event. Under all conditions tested, the heme dissociated from the myoglobin with the same, broad profile regardless of the overall ionic strength of the solution or the identity of the IL or salt used in the experiment. Additionally, the complete loss of CD signal at 409 nm is consistent with the fluorescence dequenching results indicating a complete loss of the heme from the protein (Figure 1C). 

In aggregate, the spectroscopic results indicate that the dissociation of heme from myoglobin by EBB is generally insensitive to the addition of a variety of ILs and salts, and to modest changes in the ionic strength of the solution environment. The lack of sensitivity to ionic strength is not surprising considering the hydrophobicity-driven mechanism of detergent mediated unfolding that is also the likely driver of heme displacement from the binding pocket. The range of ionic strengths tested in this work is relatively modest considering the typical ionic strengths in biological environments, so the protein stability is not likely to be significantly affected. More interestingly, the heme dissociation profile was not significantly affected by the inclusion of ILs, especially BMIBF_4_ which has been shown to destabilize myoglobin, enhancing the denaturing effects of GuHCl. The results presented in Figure 3, Figure 4 and Figure 5 do not indicate any significant impact of BMIBF_4_ (or the other ILs) on heme dissociation, indicating there is likely no additional protein denaturation caused by the inclusion of the ILs. 

### 3.3. Thermodynamic Analysis of Heme Dissociation from Myoglobin

**Figure 6:**
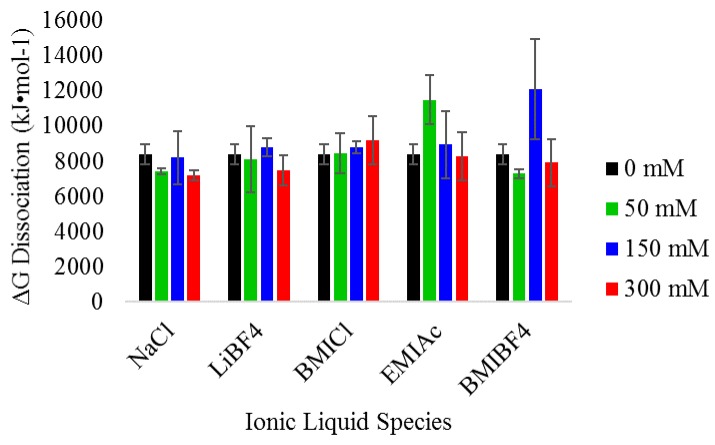
Gibbs free energy analysis of heme dissociation by EBB in the absence or presence of ionic liquids. Gibbs free energy of disassociation (ΔG_dissociation_) was calculated from the absorbance data represented in Figure 2 and Figure 3. All ΔG_dissociation_ values shown are the average of at least three individual experiments and the standard deviation of those values. An analysis of variance (ANOVA) was performed on the original data set and no statistical difference was found between any IL species or concentration of IL.

The spectroscopic experiments described allows for further quantification of the dissociation of heme from myoglobin by EBB. Assuming that the loss in heme absorbance and CD signal is attributable primarily to heme dissociation and not protein unfolding, we can use the *H*-values (Equations (1) and (3)) to compute heme-dissociation free energies. Using the data collected, the ΔG_dissociation_ was calculated for each of the IL/salt conditions tested. As reported previously for denaturation of myoglobin, the A_409_ values from the absorbance spectroscopy experiments can be used to calculate the equilibrium constant (*K_eq_*) from the transition region of unfolding curve and then the ΔG_dissociation_ can be directly calculated [42,43]. Previously, similar transitions were used to determine ΔG_unfolding_ for the complete denaturation of the protein, but in this case, the transition more accurately reflects the ΔG_dissociation_ of the heme from the protein. The values for ΔG_dissociation_ under all conditions are shown in Figure 6. The values of ΔG_dissociation_ were ~8000 kJ/mol in most cases, regardless of the IL identity or concentration. The values were compared using an ANOVA method which showed no statistical significance based on IL identity or IL concentration.

### 3.4. Critical Micelle Concentration Determination

Many reports have focused on ILs ability to form micelles and the investigation of the denaturing mechanism in relation to a micelle- or detergent-like mechanism [51]. Considering this, the first experiments performed were to ensure that the ILs used in this study did not impact the formation of micelles by monitoring the CMC in the presence of ILs. It is widely known that the CMC of some detergents, such as SDS which is common in protein unfolding studies, are dramatically affected by the ionic strength of the environment [52,53], which is a concern for IL studies and any others where ionic strength is a variable. The environmentally sensitive fluorophore (DPH) exhibits a large increase in fluorescence emission intensity when incorporated in a nonpolar environment, such as the hydrophobic core of a micelle, and thus the polarity driven intensity change has been used in many studies to monitor the formation of micelles [52].

The CMCs for EBB in the presence of varying concentrations of IL or control salts are shown in Table 1 using high IL or salt concentrations. Based on literature, three approaches were used to fit the data: onset of fluorescence increase, which represents the beginning of micelle formation, plateau of fluorescence increase which represents saturation or nearly complete micelle formation, and midpoint of the sigmoidal increase in fluorescence. In all cases, the differences between 0 mM IL or control and the highest concentration of IL or control salt are within the standard deviations of the measurements. Using the midpoint method, all of the CMCs determined were in the range between 0.95–2.6 mM while the onset of micelle formation method ranging between 0.28–0.51 mM in all cases. The results demonstrate that the ILs and salts used do not significantly affect the CMC of Empigen BB and therefore the denaturation of myoglobin. Representative spectra of DPH above and below CMC values for EBB are shown in Supplemental Figure S1.

## 4. Discussion

The interactions of ionic liquids with biomolecules is an area of great interest due to the proliferation of ILs in various electrochemical and industrial applications. The role of the employed chemical species, concentration, and mechanism of unfolding have all been areas of significant investigation. Nonetheless, there are still questions that surround the interactions, making predictive modeling difficult at this point.

Myoglobin has been a well-studied model protein over several decades. The high abundance in biological samples, commercial availability, and favorable spectroscopic properties make it an ideal system for studying protein folding and stability. Additionally, the fact that myoglobin is exclusively composed of α-helical secondary structure allows for straightforward and unambiguous interpretation of CD spectra. These studies provide a well-established baseline of behavior for myoglobin in the presence of ionic species and detergents. The work of Tang et al. investigated the role of ionic strength on myoglobin denaturing, demonstrating that ionic strength stabilized both the native conformation and the transition (T) state of myoglobin on the unfolding pathway when thermally denatured [54]. Similarly, Stigter et al. developed a model to predicted myoglobin stability over a range of ionic strength conditions, which predicts little effect of ionic strength on myoglobin stability below 0.1 M [55]. This is consistent with the results presented here for the non-IL control samples containing NaCl and the minimal effects of ILs on the unfolding transition of myoglobin. Additionally, the transition from fully folded to partially folded may involve the formation of the molten globule (MG) state that is partially stabilized by detergents [55,56,57,58]. This is consistent with the results shown in Figure 5 in which the protein retains a significant amount of secondary structure, but loses the heme absorbance characteristics. Based on these data, it is clear that the protein does not completely denature under the conditions tested here without IL, and is unlikely to completely denature with the addition of IL alone. While the ionic strength and Hofmeister series of anions have been extensively studied for effect on protein stability, myoglobin denaturation by detergents is less well investigated. There was much early work on the effect of detergents, especially cationic and anionic species, on the behavior of myoglobin and related proteins [59,60,61]. Some of the earliest work in this area was by Blauer et al. who demonstrated that the heme group of sperm whale myoglobin was removed upon treatment with the cationic detergent lauryl pyridinium chloride (LPC) [59]. More recently, Otzen and coworkers have reported on the unfolding pathway of myoglobin when denatured with cationic and anionic detergents, as well as a rhamnolipid based biosurfactant [5,62,63,64]. Their work elegantly describes the complexity of detergent denatured proteins, specifically highlighting the variety of intermediate structures that form, as well as how these structures vary based on the identity of the detergent used in the studies [5,63]. Specifically, the comparison of sodium dodecyl sulfate (SDS) and *N*-dodecyltrimethylammonium chloride (DTAC) demonstrated that the detergents destabilize certain regions of the myoglobin protein, rather than causing total denaturation [5,63]. This is consistent with the observed partial denaturation induced by EBB. Similarly, Moriyama and Takeda demonstrated that myoglobin retained a significant amount of helical content when denatured by SDS at 25 °C (~60% helical compared to ~80% in the absence of SDS), which was also true at the elevated temperature of 60 °C (~40% helical compared to ~70% in the absence of SDS) [48]. There were no reports in the literature that used EBB as a denaturing agent for myoglobin. However, one important difference between the work presented here and that of Otzen and colleagues and Moriyama and Takeda is the charge on the detergent: EBB being zwitterionic while SDS and DTAC are anionic and cationic, respectively. Most of the reports using EBB focus on the ability to solubilize protein complexes or membrane proteins [44,65,66]. The experiments presented demonstrate the first application of EBB as a denaturing agent for proteins.

The denaturation profile of myoglobin with and without ILs present show similar behavior, with only partial loss of helical content. However, this loss of helical content is concomitant with a dramatic loss of heme absorbance. These results can be interpreted in the detergent denaturing secondary structural elements near the heme binding pocket, thus disrupting the molecular contacts that allow the heme to be positioned within the protein. The preponderance of hydrophobic amino acids in the heme binding pocket provide an ideal binding site for detergent molecules, and thus provide a likely point of local structural disruption (Supplemental Figure S2). The EBB clearly induced a loss of helical content in the protein, from ~67% down to ~52% helix with 1.75 mM detergent and further to 43% helix with 8 mM detergent, as judged by several software algorithms [67]. This incomplete denaturation by detergents is consistent with the previously published work of Takeda et al. and Otzen et al., as well as with a model in which the detergents initially bind to and disrupt the heme binding site [5,48]. It is unclear if EBB will completely denature other proteins that are known to lose all secondary and tertiary structure upon detergent exposure.

There have been many previous reports of how ILs affect stability of proteins, including myoglobin, and the role of both the cation and anion in these interactions. Previous work by Fiebig et al. demonstrated the significant destabilizing effect of 150 mM and 300 mM BMIBF_4_ on myoglobin denatured by GuHCl. The same work showed a much weaker destabilizing effect of EMIAc, pointing to the role of the anion in the mechanism of protein destabilization. Similarly, Constantinescu et al. investigated the role of EMI-based ILs and the Hofmeister series on the stability of RNase, demonstrating that the anion (Tf_2_N)—was the most destabilizing anion when coupled with the EMI cation, [68]. These studies were performed under conditions with very high IL concentrations, up to 1.5 M in some cases. This is an important caveat, especially with the ions in the Hofmeister series, as it has been shown that ionic strength can have both stabilizing and destabilizing influences on α-helices, dependent on the concentration regime [69]. Notably, high concentrations of BMIBF_4_ (300 mM) will denature myoglobin over time thus the effects of low concentrations of IL are relevant and important to understand. The results presented here indicate that lower concentrations of the disruptive IL BMIBF_4_ do not have significant effects on the dissociation of heme from myoglobin. 

The mechanism of IL-based protein denaturation is also still an open question in the field. Many reports have focused on the surfactant-like properties of imidazolium-based ILs, linking the surfactant properties to the length of the alkyl chain attached to the imidazole ring [70,71,72]. Many of the literature reports on IL-protein interaction link protein stability to alkyl chain length, with increasing alkyl chain length contributing to greater destabilization of protein structures. This was demonstrated using imidazolium-based ILs by Rudolph and coworkers as well as for tetraalkyl-ammonium nitrate ILs by Summers and Flowers [73,74]. This is consistent with the surfactant-like behavior of many ILs [70,72]. Lu and coworkers recently showed the effect of the cationic component of the IL had a greater impact on destabilizing myoglobin structure than the anion, including BF_4_^−^, which was attributed to either a hydrophobic or H-bonding driven mode of action [47]. Thus, the experiments presented herein were designed to specifically test structural sensitivity to surfactant like denaturation. If the ILs act in a surfactant like manner disrupting hydrophobic contacts at the interior of the protein, the combination of IL + detergent should enhance the destabilizing effect. Previous work showed that higher concentrations of BMIBF_4_ alone do not induce myoglobin unfolding under these conditions, indicating that the enhancement of unfolding was likely driven by an additive or synergistic effect between GuHCl or urea and BMIBF_4_, which is clearly not present for the EBB interactions. However, it is important to recognize that the behavior of ILs at the lower concentrations tested here may differ when concentrations are increased as in previous reports where higher concentrations of BMIBF_4_ will denature myoglobin over longer timescales, well beyond those presented above. Nonetheless, the lack of enhancement of denaturation or heme dissociation indicates the BMIBF_4_ and the EBB may destabilize the protein and heme through different mechanisms, or perhaps acting at different sites on the myoglobin. The spectroscopic results indicate that the destabilizing effects of ionic liquids on myoglobin are more complex than a simple ion-protein interaction and are likely linked to the chaotrope and the underlying molecular mechanism by which they act.

## 5. Conclusions

Taken together and in the context of the known mechanisms of interactions between myoglobin and detergent, the EBB provides an attractive alternative to traditional cationic and anionic surfactants for protein denaturation studies. The incomplete unfolding of the protein does not prevent complete loss of the heme from the binding pocket, setting up the potential for incorporation of EBB in expedited purification of heme or apo-forms of the protein. Additionally, this work shows that ionic liquid enhanced protein denaturation is dependent on the denaturing agent used. This lack of interaction between the IL and the EBB denaturing agent provides insight into related studies of proteins and ILs. Further, it suggests that ILs may be useful in applications that require partial unfolding of proteins, selective folding/unfolding conditions, and lead to more fundamental understanding of the nature of protein-IL interactions as well as the mechanisms of IL mediated stabilization and destabilization of proteins.

## Figures and Tables

**Table 1:** Critical micelle concentration (CMC) analysis.

	Lower Linear Intersection (mM)	Inflection Point (mM)	Upper Linear Intersection (mM)
**NaCl**			
0 mM	0.38 ± 0.10	1.48 ± 0.63	1.76 ± 0.82
50 mM	0.39 ± 0.16	1.34 ± 0.57	1.51 ± 0.21
100 mM	0.42 ± 0.12	1.26 ± 0.73	1.94 ± 0.23
150 mM	0.32 ± 0.08	1.15 ± 0.46	1.89 ± 0.25
300 mM	0.28 ± 0.08	0.95 ± 0.17	2.22 ± 0.12
**LiBF_4_**			
0 mM	0.46 ± 0.03	2.61 ± 0.58	2.42 ± 0.36
50 mM	0.35 ± 0.10	2.24 ± 0.54	2.52 ± 0.38
100 mM	0.51 ± 0.14	1.94 ± 0.12	3.14 ± 0.66
150 mM	0.50 ± 0.17	1.80 ± 0.38	2.91 ± 0.96
300 mM	0.29 ± 0.06	1.80 ± 0.99	2.22 ± 0.34
**BMIBF_4_**			
0 mM	0.42 ± 0.04	2.19 ± 0.63	3.05 ± 0.91
50 mM	0.36 ± 0.92	1.91 ± 0.74	3.06 ± 0.24
100 mM	0.25 ± 0.34	1.51 ± 0.16	2.86 ± 0.57
150 mM	0.31 ± 0.06	1.46 ± 0.32	2.06 ± 0.83
300 mM	0.31 ± 0.06	1.92 ± 0.54	2.24 ± 0.65
**EMIAc**			
0 mM	0.37 ± 0.05	1.57 ± 0.46	2.93 ± 0.23
50 mM	0.37 ± 0.05	2.08 ± 0.67	2.65 ± 0.43
100 mM	0.42 ± 0.11	1.94 ± 0.46	2.47 ± 0.73
150 mM	0.45 ± 0.10	2.12 ± 0.92	2.82 ± 0.56
300 mM	0.51 ± 0.07	1.67 ± 0.26	2.34 ± 0.46

## Supplementary Materials

The following are available online at http://www.mdpi.com/2218-273X/8/4/126/s1, Figure S1: Fluorescence emission spectra of DPH in the presence and absence of IL and EBB, Figure S2: 3D structure of myoglobin (PDBID 1WLA) with hydrophobic interior highlighted.
