# Supplementary material for: Heme Dissociation from Myoglobin in the Presence of the Zwitterionic Detergent N,N-Dimethyl-N-Dodecylglycine Betaine: Effects of Ionic Liquids
Source: Biomolecules. 2018 Oct 29;8(4):126. doi: 10.3390/biom8040126 (PMC6315634; doi:10.3390/biom8040126)
Supplement: Supplementary file 1 [file biomolecules-08-00126-s001.pdf]

# Supplementary Material for Heme Dissociation from Myoglobin in the Presence of the Zwitterionic Detergent *N,N*-Dimethyl-*N*-Dodecylglycine Betaine: Effects of Ionic Liquids


**Eric M. Kohn [1,2], Joshua Y. Lee [1,2] , Anthony Calabro [1] , Timothy D. Vaden [1], and Gregory A. Caputo [1,3*]**

1 Department of Chemistry and Biochemistry, Rowan University, Glassboro NJ 08028 USA; kohne2@students.rowan.edu, leej8@students.rowan.edu, calabroa6@students.rowan.edu, vadent@rowan.edu, caputo@rowan.edu

2 Bantivoglio Honors College, Rowan University, Glassboro NJ 08028 USA;

3 Department of Molecular & Cellular Biosciences, Rowan University, Glassboro NJ 08028 USA;

* Correspondence: caputo@rowan.edu; Tel.: +01-856-256-5453


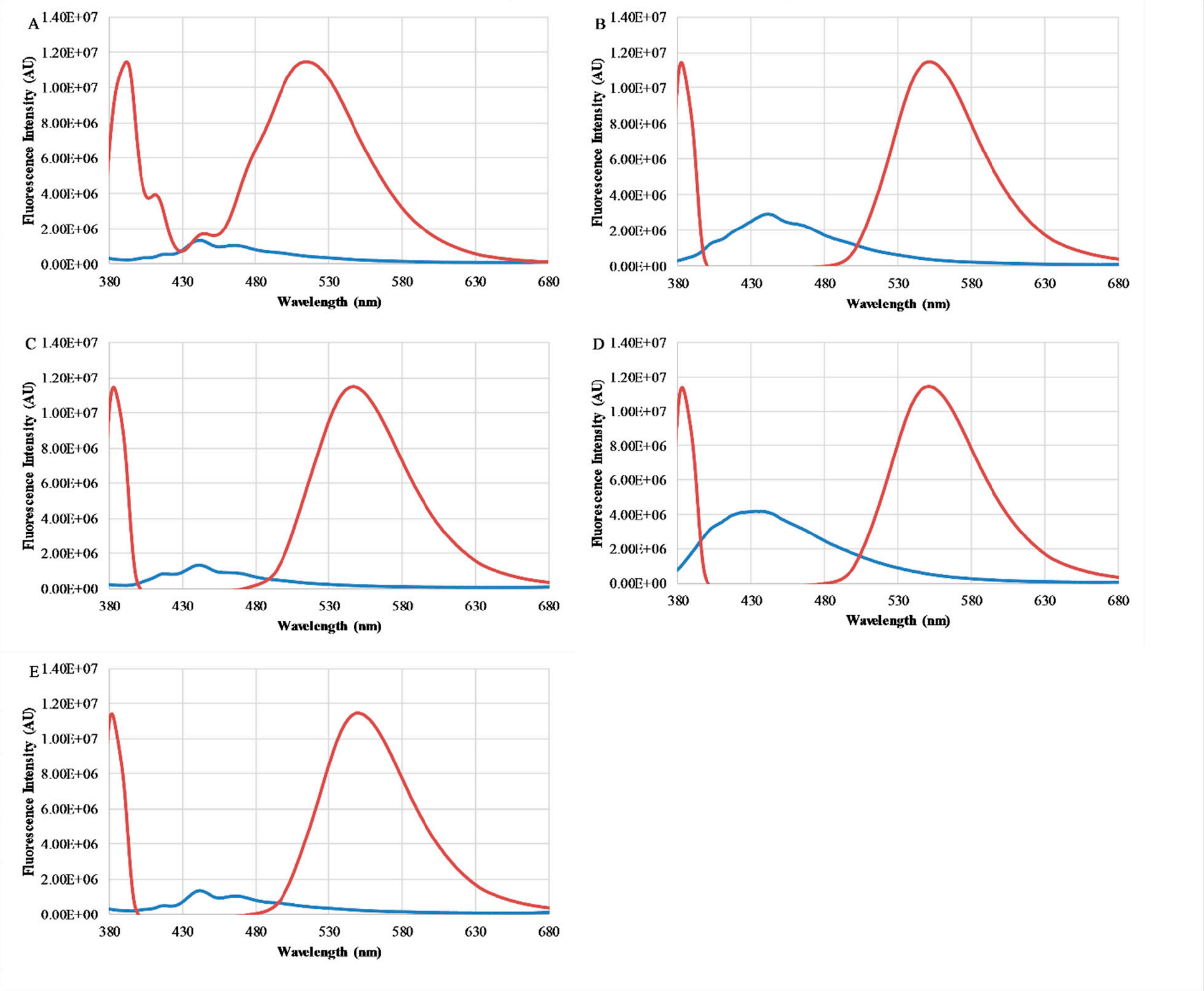


**Figure S1.** Fluorescence emission spectra of 1,6-Diphenyl-1,3,5-hexatriene (DPH) in 2mM sodium phosphate buffer, pH 7. In all panels the **blue** curve represents the emission spectrum in the absence of EBB while the red spectrum represents the emission in the presence of 8mM EBB. Each panel represents experiments done

in the presence of different ILs or controls (**A**) 2mM phosphate buffer, (**B**) 300mM NaCl, (**C**) 300mM $LiBF_4$, (**D**) 300mM $BMIBF_4$, (**E**) 300mM EMIAc.

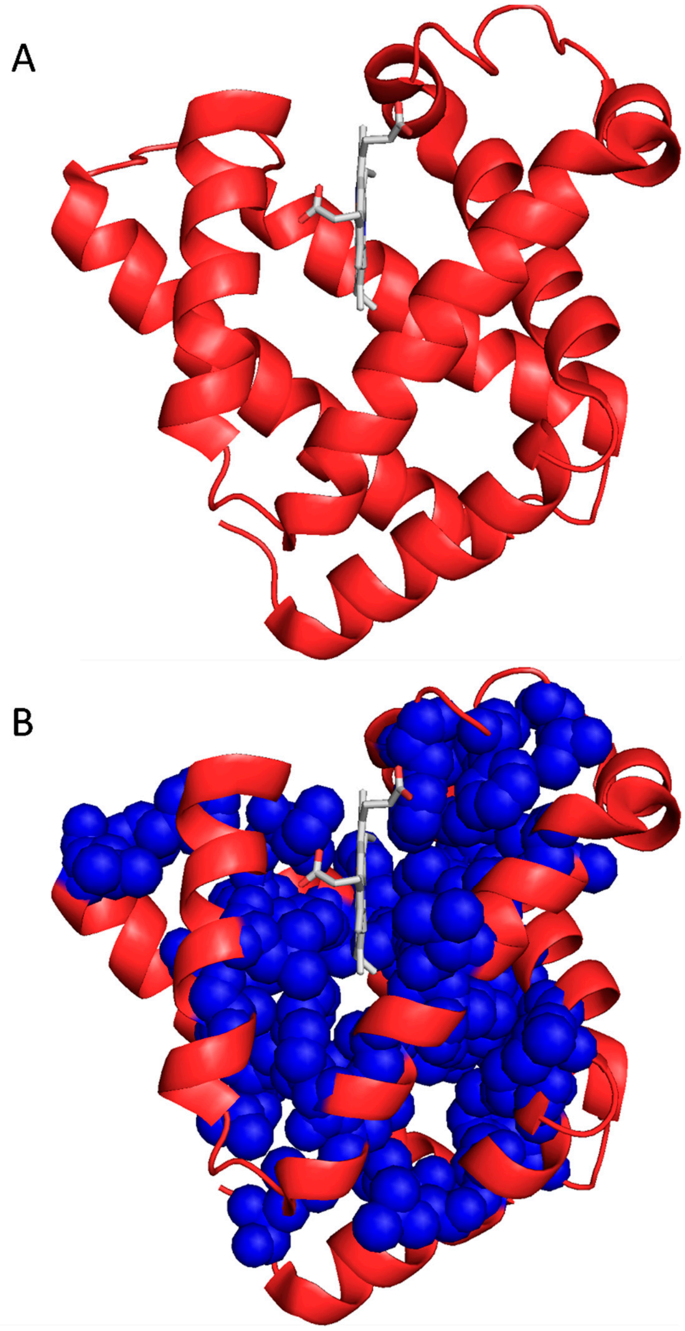


**Figure S2.** Structure of myoglobin from PDBID 1WLA. (**A**) Ribbon diagram displaying the helical character of the protein. The protein is colored red while the heme prosthetic group is gray. (**B**) Identical representation as in (A) but with hydrophobic residues shown as space-filling models in blue.
